# *Operando* visualization of water distribution hysteresis under temperature cycling in polymer electrolyte fuel cells

**DOI:** 10.1038/s41598-025-08939-7

**Published:** 2025-07-02

**Authors:** Wataru Yoshimune, Satoshi Yamaguchi, Akihiko Kato, Yoriko Matsuoka, Satoru Kato

**Affiliations:** Toyota Central R&D Labs., Inc., 41-1 Yokomichi, Nagakute, 480-1192 Aichi Japan

**Keywords:** Fuel cells, Energy

## Abstract

Polymer electrolyte fuel cells (PEFCs) face significant challenges during cold starts, where water phase transitions affect critically fuel cell performance. While previous studies have primarily focused on ice formation and melting behavior, the impact of water condensation after breaking through the freezing point remains insufficiently understood. In this study, we apply *operando* synchrotron X-ray computed tomography to visualize the transient water behavior in a PEFC under three different relative humidity (RH) conditions during a heating/cooling cycle simulating real-world cold-start conditions. The results revealed localized water flooding and hysteresis during the heating/cooling phase. A quantitative layer-by-layer analysis shows that water accumulation in each component layer strongly depends on both RH and the thermal cycle process. Moderate RH conditions promote efficient vapor-phase transport and minimize water flooding while avoiding membrane dehydration. These findings highlight water condensation as a key factor influencing cold-start performance and provide new insights into water management for more robust cold-start strategies toward next-generation PEFC systems.

## Introduction

**Fig. 1:**
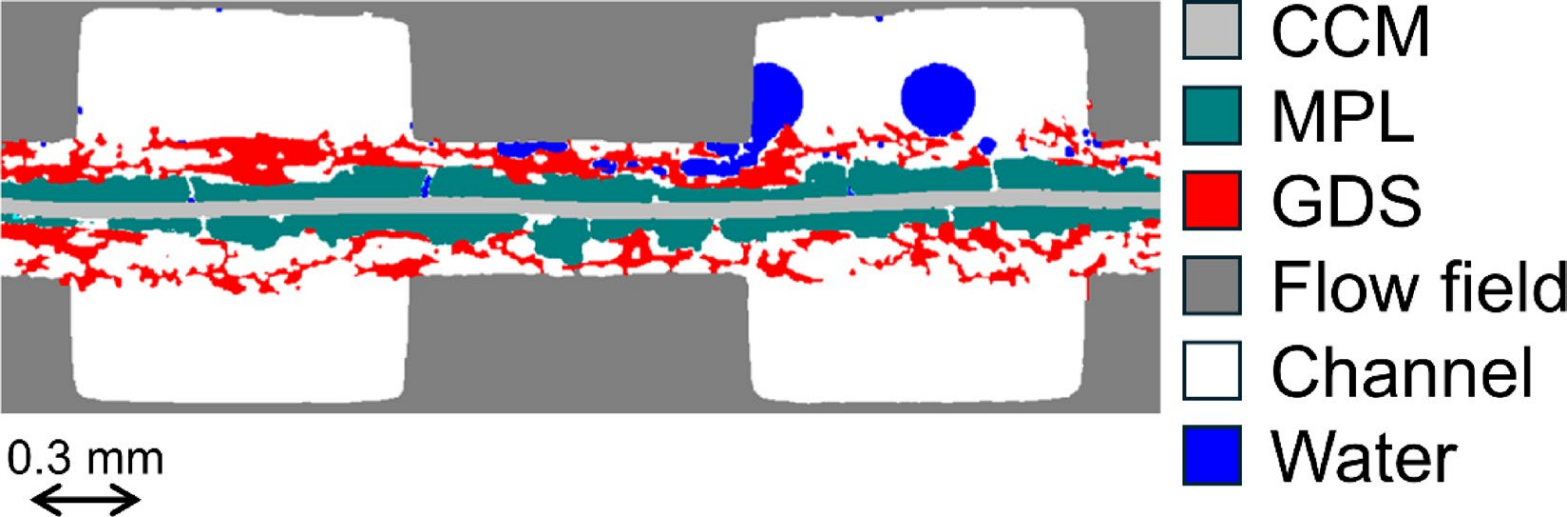
Cross-sectional visualization in a polymer electrolyte fuel cell obtained by *operando* synchrotron X-ray computed tomography (CT). The segmented image shows distinct layers: catalyst-coated membrane (CCM, light gray), microporous layer (MPL, dark green), gas diffusion substrate (GDS, red), and flow field (dark gray).

Polymer electrolyte fuel cells (PEFCs) are clean energy devices that use hydrogen as fuel and produce only heat and water as byproducts^1^. Their core component, the polymer electrolyte membrane (PEM), serves as a proton conductor, electron insulator, and gas barrier (Fig. 1). The catalyst-coated membrane (CCM) consists of catalyst layers (CLs) on both sides of the PEM, with a mixture of ionomer and Pt-based nanoparticles on carbon supports. The membrane electrode assembly (MEA) is formed by sandwiching the CCM between gas diffusion layers (GDLs), which comprise a gas diffusion substrate (GDS) and a microporous layer (MPL). The cathode CL produces water as a result of the electrochemical oxygen reduction reaction. This produced water plays a critical role in maintaining membrane hydration and proton conductivity, yet its accumulation can lead to performance losses and material degradation. The phase state of water depends strongly on the operating temperature. At elevated temperatures (typically above 60 °C), water primarily exists as vapor and is efficiently removed from the MEA, supporting stable fuel cell operation^2^. At lower temperatures, however, water vapor condenses into liquid water, which can block gas transport pathways and reduce reactant access to electrochemical reaction sites^3^. Under subzero conditions, the formation of ice at the cathode electrode can physically damage MEA components, accelerating mechanical degradation^4^.

Numerous studies have explored water transport and accumulation in PEFCs using various *operando* techniques^5,6^. In particular, *operando* imaging experiments have provided insight into water management challenges posed by water accumulation in MEAs. For example, *operando* synchrotron X-ray radiography has highlighted the effects of back-diffusion^7,8^ and oversaturation^9^ on water transport mechanisms in PEFCs and revealed high water saturation in the cathode CL^10^ and GDL^11,12^ during performance drops at high current densities. Studies have further demonstrated that water saturation in the cathode GDL depends on the polytetrafluoroethylene (PTFE) content used for waterproofing treatment^13^. Accelerated stress testing during *operando* experiments have also shown that loss of hydrophobicity in GDLs increases water saturation and leads to performance degradation^14,15^. Moreover, these techniques have identified specific water drainage pathways such as perforations^16,17^cracks^18^ large pores^19^ and percolation networks^20^ within GDLs. Flow field design using *operando* neutron radiography has also shown promise for improved water management in PEFCs^21−23^.

With the global commercialization of fuel cell electric vehicles, improving cold-start capability and performance has become increasingly critical. Recent studies using fast *operando* synchrotron X-ray computed tomography (CT) have revealed that the presence of supercooled water significantly impacts on cold-start behavior^24,25^ and pre-purging improves cold-start capability by increasing the allowable water content in the MEA^26^. *Operando* neutron imaging studies have shown that freezing events in PEFCs occur randomly and are influenced by GDL properties^27^ cell size^28^ mechanical stress^29^ or residual water^30^ resulting in high pressure drops at the cathode electrode^31^. Additionally, *operando* neutron studies have identified cathode CL freezing as a key factor in cold-start performance during rapid cold starts^32^. Neutron wavelength analysis enables the differentiation of water and ice phases^33−37^. Energy-resolved *operando* neutron imaging has demonstrated that partial freezing can trigger total freezing in large-sized PEFCs^38^.

Past cold-start research has primarily focused on ice nucleation and thawing phenomena. However, less attention has been given to water flooding caused by water condensation above the freezing point. It is worth noting that commercial fuel cell stacks employ circulating coolant systems to regulate stack temperature^39^. During cold starts, however, coolant temperature fluctuates due to the competing effects of internal heating (from power generation) and external cooling (from subzero ambient environment)^40−42^. While thermostatically controlled systems have been demonstrated to mitigate these temperature oscillations^43^, their effect on internal water distribution remains unclear.

In this study, we address this gap by employing *operando* synchrotron X-ray CT to visualize transient water behavior in PEFCs. To improve the reproducibility and generalizability of the results, we conducted a set of experiments under three different relative humidity (RH) conditions during controlled heating/cooling cycles.

## Results and discussion

### Electrochemical Performance

**Fig. 2:**
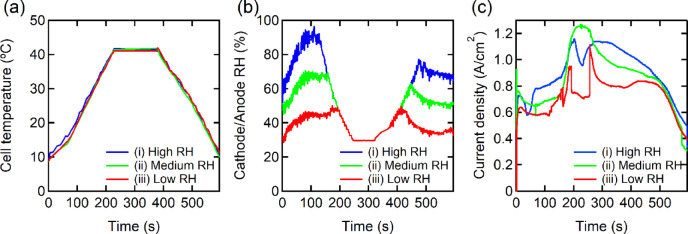
Fuel cell performance during *operando* synchrotron X-ray CT imaging under three target relative humidity (RH) conditions: (i) high RH (80%, blue), (ii) medium RH (60%, green), and (iii) low RH (40%, red). (**a**) Cell temperature profile showing a thermal cycle from 5 °C to 40 °C and back to 5 °C. (**b**) RH at both the cathode and anode inlets. (**c**) Current density response under potentiostatic operation at 0.1 V.

 To investigate the impact of RH on water distribution and electrochemical performance during transient temperature changes, we performed *operando* measurements under three target RH levels: high RH (80%), medium RH (60%), and low RH (40%). Figure 2 summarizes the temporal profiles of cell temperature, RH, and current density under these conditions as measured during *operando* synchrotron X-ray CT experiments. As shown in Fig. 2a, all tests followed nearly identical temperature profiles: the cell was heated from 5 °C to 40 °C at a rate of 1 °C every 6 s, and subsequently cooled back to 5 °C at the same rate. The temperature cycle and the ramp rate were selected to mimic the rapid thermal transients typically observed during cold-start conditions in automotive fuel cell stacks^39^. The RH profiles at the cathode/anode inlet (Fig. 2b) clearly reflect differences in humidification below 30 °C, with condition (i) exhibiting the highest RH and (iii) the lowest during both heating and cooling phases. The *operando* cell was operated in potentiostat mode at a constant voltage of 0.1 V. The electrochemical performance, represented by current density (Fig. 2c), exhibited RH-dependent behavior. Although the trends were not fully strictly systematic, condition (ii) showed the highest peak current density, exceeding 1.2 A/cm^2^, while conditions (i) and (iii) showed signs of performance degradation and fluctuation. Notably, the current density did not follow the same trajectory during the heating and cooling phases, indicating a hysteresis effect likely caused by asymmetric water accumulation and removal within the MEA. This observation suggests that RH affects not only instantaneous cell performance but also the transient water balance. Previous studies have attributed performance hysteresis to the slow change in ohmic resistance caused by the gradual water uptake of the PEM or the ionomer in the CL, as well as to the oxygen transport resistance originating from the ionomer adsorption at the ionomer/Pt interface^44^. Park et al. have claimed that water accumulation, particularly under high current densities, plays a dominant role in the performance hysteresis^45^. Since the gradual water uptake of the PEM and CL occurs over several minutes, the second-scale hysteresis observed here is likely due to rapid water accumulation in the MEA. To validate this hypothesis, we performed further analysis of water distribution in each cathode-side component of the MEA.

### Water Saturation

**Fig. 3:**
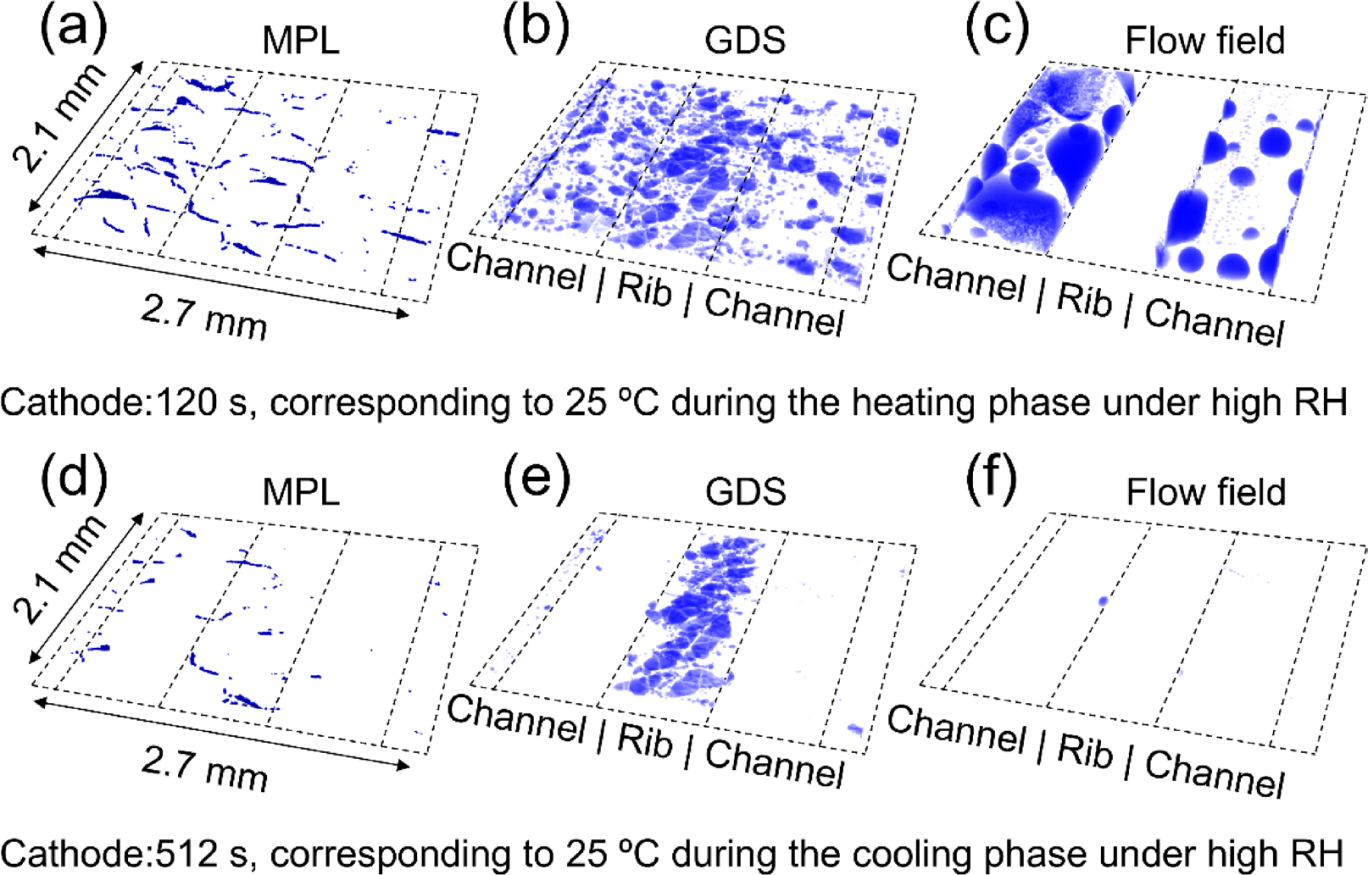
Three-dimensional water visualization in different cathode-side components, obtained from *operando* synchrotron X-ray CT under high RH. (a–c) Water distribution at 120 s, corresponding to 25 °C during the heating phase; (d–f) water distribution at 512 s, corresponding to 25 °C during the cooling phase. Components shown include: (**a**, **d**) water localized in cracks within the MPL, (**b**, **e**) water accumulation in the GDS, and (**c**, **f**) discrete water droplets formed in the flow channel.

 In a representative cross-sectional image of the PEFC (Fig. 1), liquid water was clearly observed on the cathode electrode, extending from cracks within the MPL to the flow channel through the GDS. In contrast, no detectable water signal was observed on the anode side throughout the measurements. Figure 3 shows the water distribution along the stacking direction of the MEA at a representative time point (120 s, corresponding to 25 °C during both heating and cooling phases) under high RH. Liquid water was localized within some cracks in the MPL (Fig. 3a, d), while water accumulation in the GDS was observed mainly in areas compressed by the flow field (Fig. 3b, e). Discrete water droplets were also visualized in the flow channels (Fig. 3c, f). Due to the spatial resolution limitations of synchrotron X-ray CT, it was impossible to visualize water clusters residing in the pores of the MPL. Cracks in the MPL promote water transport away from the cathode CL, thereby preventing flooding at the cathode CL/MPL interface^16–20^. However, water droplets within the nanopores of the CL and water film present at the interface remained invisible due to the strong X-ray absorption of platinum^10^. During the heating phase (Fig. 3a–c), significant water accumulation was observed in the GDS and flow field, along with distinct water presence in cracks within the MPL. In contrast, during the cooling phase (Fig. 3d–f), water volumes in all regions were noticeably reduced, particularly in the GDS where only accumulated water remained under the rib. These differences at the same temperature indicate a hysteresis effect, likely driven by phase transition asymmetry. This observation highlights the importance of transient temperature profiles in determining the water saturation level.

**Fig. 4:**
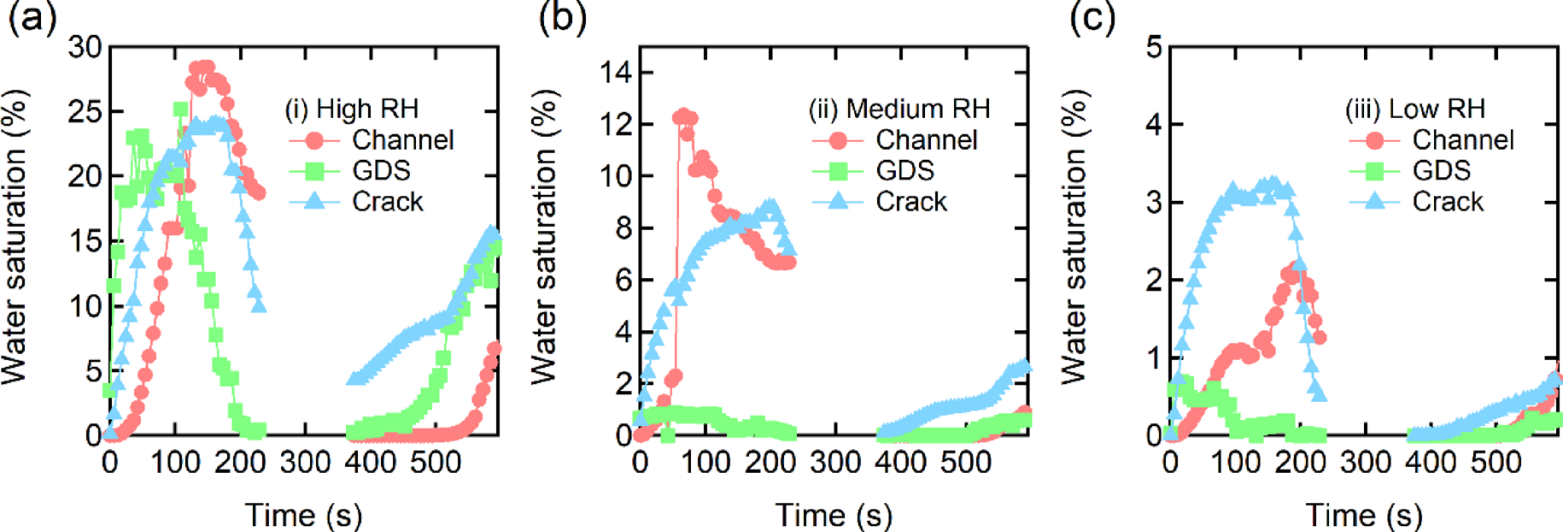
Time evolution of water saturation in each cathode-side component of the membrane electrode assembly under three RH conditions: (**a**) high RH, (**b**) medium RH, and (**c**) low RH. Water saturation is quantified for the flow channel (light red circles), GDS (light green squares), and cracks within the MPL (light blue triangles). All measurements were conducted during a controlled thermal cycle from 5 °C to 40 °C and back under potentiostatic operation. A gap is observed in all curves between 250 s and 320 s, corresponding to a programmed pause in X-ray CT scanning to reconfigure the system from the heating to the cooling phase.

Figure 4 shows the time evolution of water saturation in each cathode-side component of the MEA (namely, the flow channel, GDS, and cracks within the MPL) under three RHs. During the heating phase, water saturation increased significantly in all regions under high RH (Fig. 4a), with peak values exceeding 25% in the flow channel and 20% in the GDS and MPL cracks. Under medium RH (Fig. 4b), overall water saturation was suppressed. Water accumulation in the GDS decreased substantially, and water saturation in both the MPL cracks and the flow channel remained below 15%. This suggests that a moderate RH level effectively balances water accumulation and removal, enabling stable operation without excessive flooding or membrane dehydration. In particular, the reduced water saturation in the GDS promotes air transport by maintaining sufficient gas diffusivity^46^. Under low RH (Fig. 4c), water saturation further decreased across all regions, including the MPL cracks and the flow channel. Although this condition minimizes the risk of air-blocking water accumulation, it may also increase the likelihood of membrane dehydration and associated performance losses.

During the cooling phase, an increase in water saturation was observed in all RHs, particularly in the MPL cracks. This increase is attributed to enhanced condensation at lower cell temperatures. In the high RH case, water saturation in the flow channel increased again at the end of the cooling phase. In contrast, medium and low RHs showed more moderate or minimal changes, respectively. Overall, the water accumulation patterns during heating and cooling were asymmetric. Produced water tended to accumulate more rapidly and extensively during heating due to enhanced condensation at high RH, while slower removal and ongoing water condensation during cooling contributed to persistent water saturation. These findings highlight that both heating and cooling phases significantly affect water behavior.

**Fig. 5:**
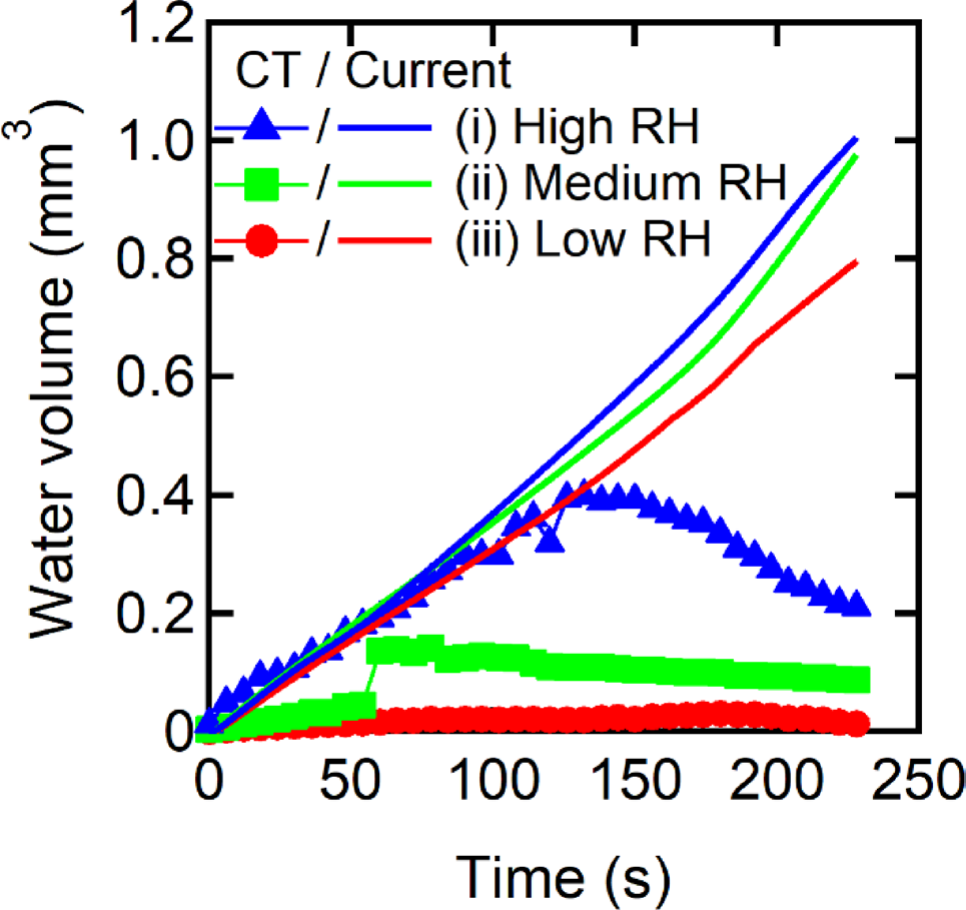
Comparison of observed water volume at the cathode electrode and calculated water production from current under (i) high RH (blue), (ii) medium RH (green), and (iii) low RH (red) conditions during the heating phase.

To distinguish between liquid-phase and vapor-phase transport, we evaluated the water balance by comparing the observed water volume at the cathode electrode with the theoretical water production calculated from the current (Fig. 5). Under high RH, the measured water volume closely matched the calculated value, indicating that water was primarily transported in the liquid phase. In contrast, medium and low RHs showed substantial discrepancies, suggesting more effective water removal via vapor-phase transport. In addition, a decline in water accumulation was observed around 150 s, particularly under high RH. This rapid decrease is attributed to enhanced evaporation of accumulated water as the cell temperature increases, marking a transition from liquid-phase to vapor-phase dominant transport. This transition temperature region reflects a shift in the prevailing water transport mechanism driven by phase change dynamics. In Fig. 4, this decrease was clearly resolved across individual cathode-side components: a pronounced drop in water saturation was observed in the GDS and flow field, while only a modest change was observed in cracks within the MPL. This layer-resolved behavior highlights the delay in the prevailing water transport mechanism driven by phase change dynamics.

To interpret the observed hysteresis in water saturation and fuel cell performance, it is essential to consider the thermodynamic asymmetry between heating and cooling. During heating, evaporation absorbs latent heat, whereas during cooling, water condensation releases it. This hysteresis is not merely due to transport lag, but is fundamentally linked to energy exchange and phase equilibrium within the MEA. Such effects have been noted in previous studies on phase-change phenomena in porous media^26^. While earlier studies have demonstrated both operability under thermal transients and analyzed water saturation using synchrotron X-ray^27^ or neutron imaging^28^, our study distinguishes itself by capturing dynamic water condensation behavior above the freezing point under varying RHs. The identification of hysteresis linked to water condensation dynamics offers new insight into water management during cold-start transients.

Finally, to contextualize the significance of our findings, we discuss the key transport processes and their characteristic time constants governing water and heat behavior during cold-start transients. Relaxation of proton conduction within the PEM typically occurs over several minutes, due to gradual water uptake and reorganization of the ionomer structure^44^. Changes in oxygen transport resistance due to ionomer swelling and wetting occur over tens of seconds to several minutes^45^. In contrast, evaporation and water condensation processes within the GDS take place on a faster timescale, generally ranging from one to ten seconds depending on RH and material porosity^12^. Liquid water removal from the GDS to the flow channel typically occurs within one to five seconds, although this strongly depends on local compression and the presence of cracks or transport pathways^19^. The alignment of these timescales with our synchrotron X-ray CT frame rate (every 2 s) enables the transient dynamics to be resolved *operando*. This temporal resolution, combined with spatial layer analysis, provides a deeper understanding of the coupled processes influencing cold-start behavior.

## Conclusion

This study provides the first *operando* layer-resolved visualization of water condensation dynamics above the freezing point in PEFCs during cold-start-like thermal transients. By combining fast synchrotron X-ray CT imaging with a forced cooling setup, we demonstrated that water behavior was not only RH-dependent but also exhibited thermal hysteresis between the heating and cooling phases. Under high RH, we observed a rapid change in water saturation level due to the transition of water transport mechanism driven by phase change dynamics. Importantly, this hysteresis did not arise from thermal lag, but rather from fundamental thermodynamic asymmetries in latent heat exchange and phase transition mechanisms. These findings extend the current understanding of cold-start water management by highlighting vapor–liquid condensation, in addition to classical ice-thaw transitions, as a critical limiting factor. Moreover, these insights provide a revised framework for understanding water transport in PEFCs under dynamic conditions and suggest new directions for next-generation stack control strategies targeting robust cold-start performance.

## Methods

### Materials

 A CCM was fabricated by hot-pressing the electrode decals onto both sides of a Nafion membrane (NR211, Chemours, USA). Pt nanoparticles supported by Vulcan carbon (Pt/C, TEC10V30E, 29.3 wt% Pt, Tanaka Kikinzoku Kogyo, Japan), Nafion dispersion (D2020CS, Chemours, USA), ethanol (99.5%, FUJIFILM Wako Pure Chemical Corp., Japan), and deionized water were used to prepare a catalyst ink. Pt/C (300 mg) and Nafion ionomer (767 mg) were dispersed in ethanol/deionized water (1/3, *v*/*v*, 43.5 mL) to obtain a catalyst ink with a solid content of 10 wt%. A glass vial containing this mixture was placed in an ultrasonic bath filled with cold water and sonicated for 10 min^47^. The ink was applied to a PTFE sheet and dried under vacuum at 120 °C for 10 min. The obtained CLs were transferred to both sides of the Nafion membrane by annealing at 120 °C for 10 min^48^. For both the cathode and anode, the Pt loading in the 0.162 cm^2^ CCM was 1.0 mg/cm^2^. A GDS with an MPL was used for the GDL (Sigracet 22BB, SGL Carbon, Germany). The MEA with ethylene-propylene-dienemonomer rubber gaskets was compressed using two electrically conductive flow fields to fabricate an *operando* cell. The flow fields had two straight parallel flow channels with depths, lengths, and widths of 0.3, 1.0, and 0.8 mm, respectively.

### *Operando* Synchrotron X-ray CT

**Fig. 6:**
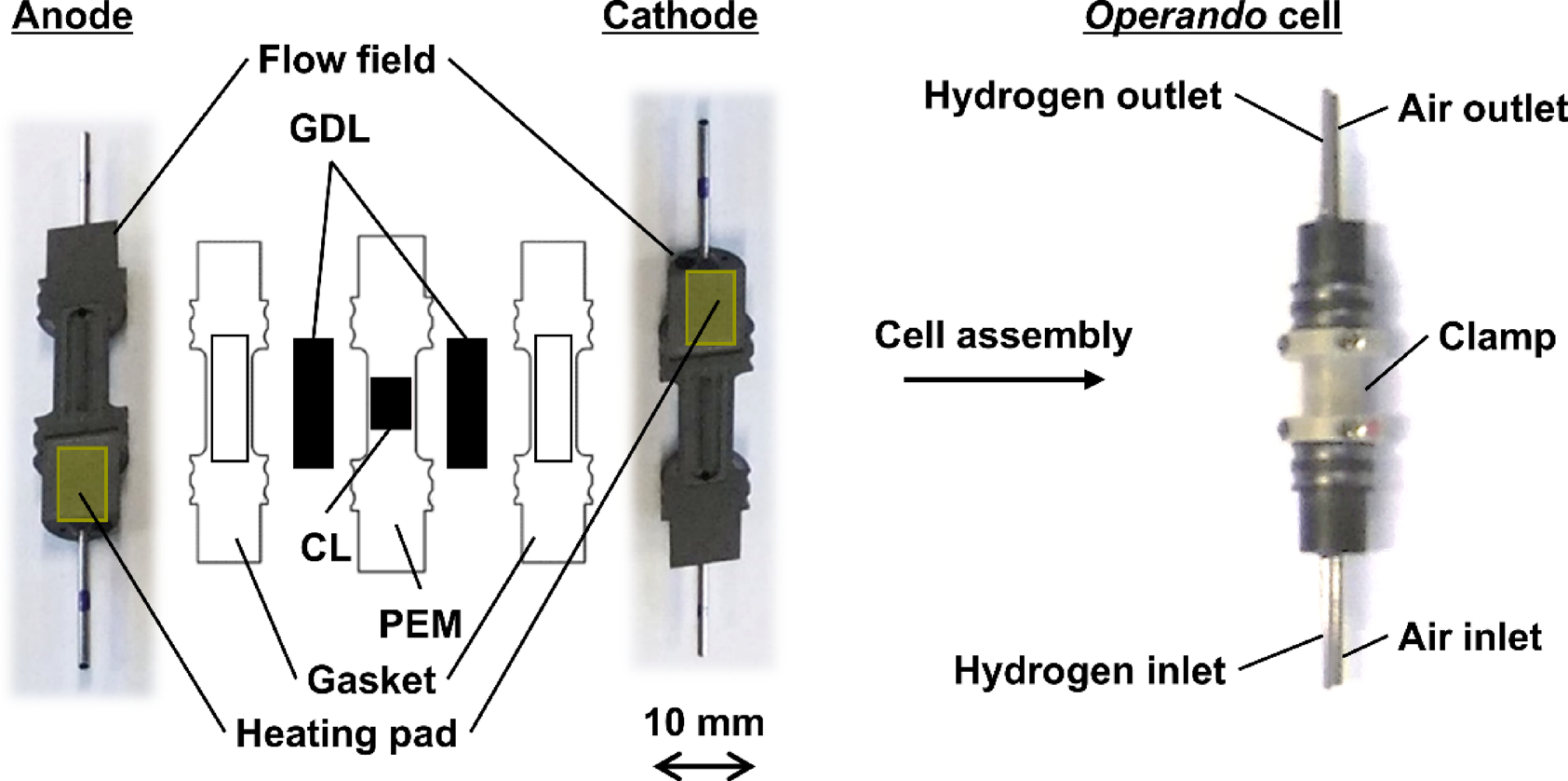
Schematic and photograph of the *operando* fuel cell used for synchrotron X-ray CT. The left panel shows an exploded view of the cell components, including the flow field plates, gas diffusion layers (GDLs), catalyst layers (CLs), polymer electrolyte membrane (PEM), gaskets, and heating pads. The right panel shows the fully assembled *operando* cell, with labeled gas inlets and outlets for hydrogen and air.

**Fig. 7:**
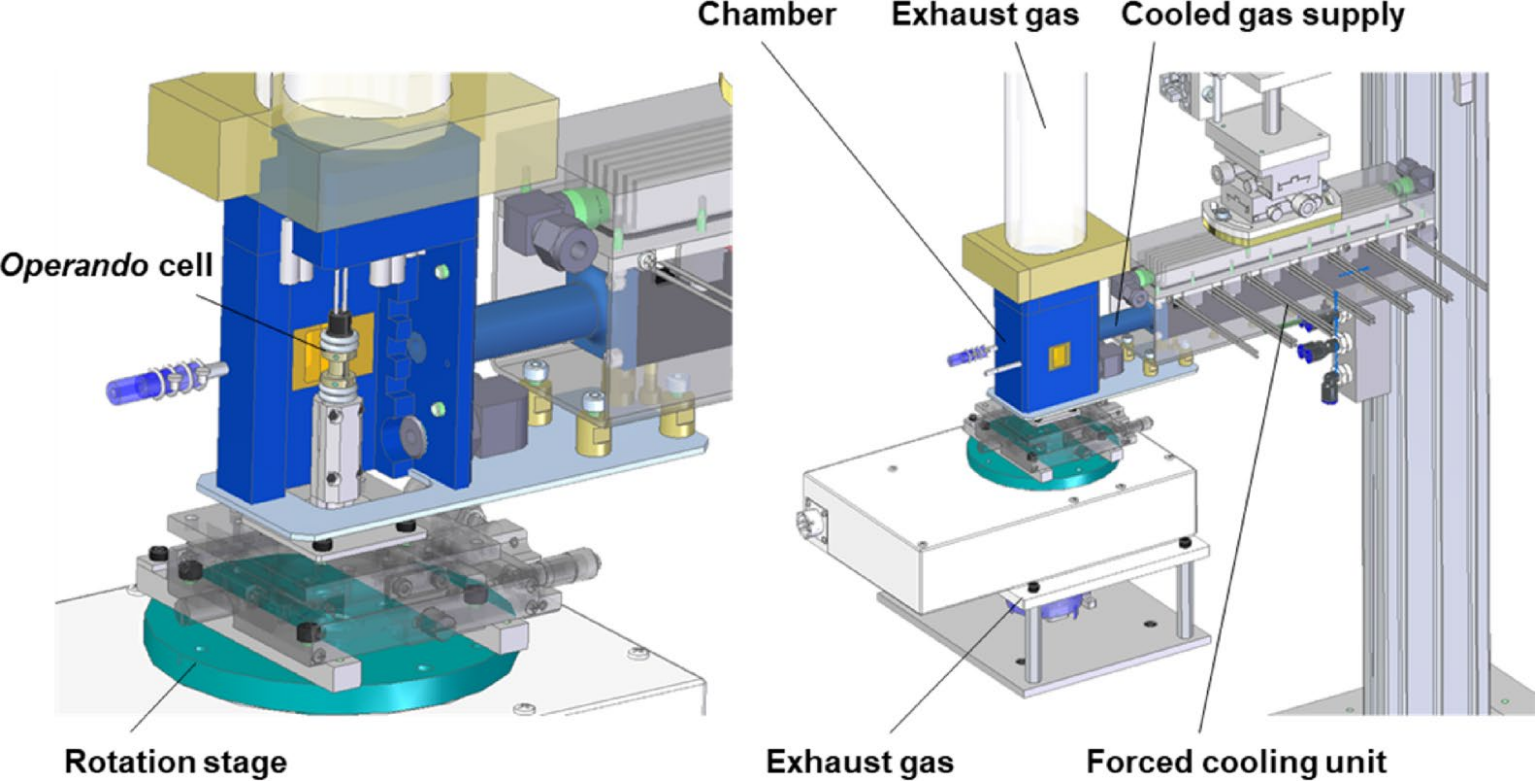
Schematic of the *operando* synchrotron X-ray CT system equipped with a forced cooling unit. The figure was generated using Siemens Solid Edge 2023 (version 2210.0010)^49^. The *operando* cell is mounted on a high-speed rotation stage within a sealed chamber to enable imaging during fuel cell operation. A cooled gas supply and forced convection cooling unit enable rapid and controllable temperature cycling of the *operando* cell, down to subzero conditions. Exhaust lines are integrated to remove reaction and cooling gases. This setup allows for *operando* observation of water dynamics under realistic cold-start and transient thermal conditions.

Our previous *operando* synchrotron X-ray CT setup^19^ was updated prior to the present work with an additional function for studying PEFCs (Fig. 6)^25,26^. A high-speed rotary stage (Kohzu Precision, EM200-11) was used for X-ray CT scanning. For *operando* measurements, slip rings and rotary joints were used for wiring and gas piping, respectively. These features enabled high-speed X-ray CT measurements with a rotational speed and accuracy of 1800°/s and 1 μm, respectively. The fuel cell was surrounded by a resin chamber to prevent condensation and increase cooling efficiency. Dry air supplied from a compressor was cooled down to − 30 °C using a Peltier device and blown into the chamber (Fig. 7). The temperature was monitored at the top and bottom of the cell, and their average was defined as the cell temperature. The cell temperature was successfully reduced to a minimum of − 20 °C using our forced cooling unit. The fabricated cell was conditioned at 40 °C and RH 30% under 200 cm^3^/min in an air environment at the cathode and 100 cm^3^/min in a hydrogen environment at the anode without back pressure. The details of the break-in process are described in our previous study^19^. Following the break-in procedure, the fuel cell was cooled to 5 °C under the same gas flow rate. Subsequently, the fuel cell was operated at a constant voltage of 0.1 V with temperature control for *operando* measurements. In this study, RH was controlled by mixing humidified gas (dew point 18 °C) and dry gas, without accounting for water produced by the electrochemical reaction. The mixing ratio was continuously adjusted to match the target water vapor partial pressure, which was calculated based on the cell temperature corresponding to the desired RH level. The three target RH levels were high RH (80%), medium RH (60%), and low RH (40%). This control procedure was consistently applied across all RH conditions and during both the heating and cooling phases, ensuring symmetrical experimental conditions. At the midpoint of the temperature cycle, the RH values overlapped among all conditions (Fig. 2b), reflecting the state in which 100% humidified gas was supplied and RH varied in response to changes in cell temperature. During the heating phase, the actual RH values closely matched the setpoint. However, during the cooling phase, particularly in the low-temperature region, the RH tended to fall below the target. This behavior indicates the inherent limitations of our RH control method, where small differences in water vapor partial pressure can result in large deviations in RH levels. The *operando* synchrotron X-ray CT measurements were performed on a Toyota beamline (BL33XU) at the SPring-8 facility^50^ with an X-ray energy of 20 keV and a pixel size of 2.96 μm. The offset scan in the phase contrast mode was acquired using the on-the-fly method with 1,500 projections during a 360° continuous rotation in 2 s. During the thermal cycling, X-ray CT scanning was intentionally paused between 250 s and 320 s to allow for reconfiguration of our system from the heating phase to the cooling phase. This pause was required due to technical constraints associated with the forced cooling unit. Throughout this interval, the cell temperature and RH were maintained at stable levels, but no imaging data were acquired. Therefore, a gap appears in the plotted saturation curves in Fig. 4.

### Thermal response

 The total heat generated during operation was 0.12 W/cm^2^ at the peak current density (1.2 A/cm^2^) and a cell voltage of 0.1 V, corresponding to a total heat output of 0.02 W for the effective active area of 0.162 cm^2^. The total heat capacity of the cell was estimated to be ~ 2.54 J/K, based on each component including the PEM, CLs, GDLs, and especially the graphited flow field plates. This corresponds to a required heat generation rate of about 0.42 W. The estimate clearly indicates that the cell temperature was governed primarily by external heating rather than self-heating. To further evaluate the thermal responsiveness during the programmed temperature change, we estimated the thermal diffusion times of its major components. The carbon paper type GDL (~ 200 μm thick) has a thermal diffusivity of ~ 4.4 × 10⁻^6^ m^2^/s, resulting in a rapid thermal response of ~ 9 ms. The graphite flow field plates, evaluated over a 10 mm characteristic length (corresponding to the distance from thermocouple placement), exhibit a much higher thermal diffusivity of ~ 7.1 × 10⁻^5^ m^2^/s, yielding a diffusion time of ~ 1.4 s. These values, consistent with thermal conductivity from Csoklich et al.^51^, confirm that both the GDL and flow field respond well within the imposed heating/cooling rate. Thus, the entire cell was considered thermally quasi-equilibrated during our experiments.

### Post-processing for imaging data

 Image reconstruction was performed using a software package provided by the Japan Synchrotron Radiation Research Institute^52^and rotation correction was performed using ImageJ software^53^. The obtained phase contrast X-ray CT images were processed using Amira software. Ring artifact reduction is first executed. Next, the flow field region was extracted using a watershed segmentation method, and the CCM region was identified using a threshold segmentation method. The GDS was then determined using a top-hat segmentation method. Finally, the MPL was indexed using a watershed segmentation method. The remaining portion was recognized as the flow channel. An additional water region was assigned to the dry image by comparing the images before and after power generation. As a result, six-phase segmentation was achieved under *operando* conditions. The segmented images were visualized using GeoDict software^54^. Based on the representative elementary volume analysis of GDLs reported by Zenyuk et al.^55^, an analytical area of 5.67 mm^2^ was selected for water quantification to ensure statistical representativeness.

## Data Availability

Data are available upon reasonable request from the corresponding author.

## References

[1] Kodama, K., Nagai, T., Kuwaki, A., Jinnouchi, R. & Morimoto, Y. Challenges in applying highly active Pt-based nanostructured catalysts for oxygen reduction reactions to fuel cell vehicles. *Nat. Nanotechnol*. **16**, 140–147. 10.1038/s41565-020-00824-w (2021). 33479539 10.1038/s41565-020-00824-w

[2] Shum, A. D. et al. Investigating phase‐change‐induced flow in gas diffusion layers in fuel cells with X‐ray computed tomography. *Electrochim. Acta* **256**, 279–290, 10.1016/j.electacta.2017.10.012 (2017).

[3] Liu, Q., Lan, F., Chen, J., Zeng, C. & Wang, J. A review of proton exchange membrane fuel cell water management: membrane electrode assembly. *J. Power Sources*. **517**, 230723. 10.1016/j.jpowsour.2021.230723 (2022).

[4] Liang, J., Fan, L., Du, Q., Yin, Y. & Jiao, K. Ice formation during PEM fuel cell cold start: Acceptable or not? *Adv. Sci.* **10**, 2302151. 10.1002/advs.202302151 (2023). 10.1002/advs.202302151 PMC10460847 37344346

[5] Lang, J. T. et al. I.V. X-ray tomography applied to electrochemical devices and electrocatalysis. *Chem. Rev.* **123**, 9880–9914. 10.1021/acs.chemrev.2c00873 (2023). 37579025 10.1021/acs.chemrev.2c00873 PMC10450694

[6] Yoshimune, W. Multiscale characterization of polymer electrolyte fuel cells elucidated by quantum beam analysis. *Bull. Chem. Soc. Jpn.* 97, uoae046, DOI:1 (2024). 10.1093/bulcsj/uoae046

[7] Yoshimune, W. et al. 3D water management in polymer electrolyte fuel cells toward fuel cell electric vehicles. *ACS Energy Lett.* **8**, 3485–3487. 10.1021/acsenergylett.3c01096 (2023).

[8] Kato, A. et al. Operando X-ray radiography of liquid water distribution in 100 mm polymer electrolyte fuel cell channels. *Electrochem. Commun.* **165**, 107772. 10.1016/j.elecom.2024.107772 (2024).

[9] Kato, A., Kato, S., Yamaguchi, S., Suzuki, T. & Nagai, Y. Mechanistic insights into water transport in polymer electrolyte fuel cells with a variation of cell temperature and relative humidity of Inlet gas elucidated by operando synchrotron X-ray radiography. *J. Power Sources*. **521**, 230951. 10.1016/j.jpowsour.2021.230951 (2022).

[10] Yoshimune, W., Kato, A., Hayakawa, T., Yamaguchi, S. & Kato, S. Liquid water visualization in the Pt-loading cathode catalyst layers of polymer electrolyte fuel cells using operando synchrotron X-ray radiography. *Adv. Energy Sustain. Res.* **5**, 2400126. 10.1002/aesr.202400126 (2024).

[11] Matsui, H., Ohta, T., Nakamura, T., Uruga, T. & Tada, M. *In situ* 3D X-ray imaging of water distribution in each layer of a membrane electrode assembly of a polymer electrolyte fuel cell. *Phys. Chem. Chem. Phys.* **26**, 15115–15119. 10.1039/D4CP00728J (2024). 38592673 10.1039/d4cp00728j

[12] Chen, Y. C. et al. On the water transport mechanism through the microporous layers of *operando* polymer electrolyte fuel cells probed directly by X-ray tomographic microscopy. *Energy Adv.* **2**, 1447–1463. 10.1039/D3YA00189J (2023). 38014390 10.1039/d3ya00189j PMC10500626

[13] Yoshimune, W., Kato, A., Hayakawa, T., Yamaguchi, S. & Kato, S. Comprehensive analysis of wettability in waterproofed gas diffusion layers for polymer electrolyte fuel cells. *ACS Appl. Mater. Interfaces*. **16**, 36489–36497. 10.1021/acsami.4c07867 (2024). 38965821 10.1021/acsami.4c07867 PMC11863250

[14] White, R. T. et al. Four-dimensional joint visualization of electrode degradation and liquid water distribution inside operating polymer electrolyte fuel cells. *Sci. Rep.* **9**, 1843. 10.1038/s41598-018-38464-9 (2019). 30755635 10.1038/s41598-018-38464-9 PMC6372714

[15] Yoshimune, W., Kato, A., Hayakawa, T., Yamaguchi, S. & Kato, S. Simultaneous accelerated stress testing of membrane electrode assembly components in polymer electrolyte fuel cells. *Npj Mater. Degrad.* **8**, 106. 10.1038/s41529-024-00524-z (2024).

[16] Alink, R. et al. The influence of porous transport layer modifications on the water management in polymer electrolyte membrane fuel cells. *J. Power Sources*. **233**, 358–368. 10.1016/j.jpowsour.2013.01.085 (2013).

[17] Haußmann, J. et al. Synchrotron radiography and tomography of water transport in perforated gas diffusion media. *J. Power Sources*. **239**, 611–622. 10.1016/j.jpowsour.2013.02.014 (2013).

[18] Sasabe, T., Deevanhxay, P., Tsushima, S. & Hirai, S. Soft X-ray visualization of the liquid water transport within the cracks of micro porous layer in PEMFC. *Electrochem. Commun.* **13**, 638–641. 10.1016/j.elecom.2011.03.033 (2020).

[19] Nagai, Y. et al. Improving water management in fuel cells through microporous layer modifications: Fast *operando* tomographic imaging of liquid water. *J. Power Sources*. **435**, 226809. 10.1016/j.jpowsour.2019.226809 (2019).

[20] Dörenkamp, T., Büchi, F. N., Schmidt, T. J. & Eller, J. Exploring chances and limitations of high resolution 3D-printing for guided water percolation in gas diffusion layers of polymer electrolyte fuel cells. *InterPore J.* **1**, IPJ271124–IPJ271123. 10.69631/ipj.v1i3nr43 (2024).

[21] Nasu, M. et al. Neutron imaging of generated water inside polymer electrolyte fuel cell using newly-developed gas diffusion layer with gas flow channels during power generation. *J. Power Sources*. **530**, 231251. 10.1016/j.jpowsour.2022.231251 (2022).

[22] Wu, Y. et al. Water management and mass transport of a fractal metal foam flow-field based polymer electrolyte fuel cell using *operando* neutron imaging. *Appl. Energy*. **364**, 123204. 10.1016/j.apenergy.2024.123204 (2024).

[23] Trogadas, P. et al. A nature-inspired solution for water management in flow fields for electrochemical devices. *Energy Environ. Sci.* **17**, 2007–2017. 10.1039/D3EE03666A (2024).

[24] Mayrhuber, I., Marone, F., Stampanoni, M., Schmidt, T. J. & Buchi, F. N. Fast X-ray tomographic microscopy: Investigating mechanisms of performance drop during freeze starts of polymer electrolyte fuel cells. *ChemElectroChem* **2**, 1551–1559. 10.1002/celc.201500132 (2015).

[25] Sabharwal, M. et al. Understanding the effect of feed gas humidity on the freeze start behavior of polymer electrolyte fuel cells. *J. Electrochem. Soc.* **168**, 114512. 10.1149/1945-7111/ac37ed (2021).

[26] Sabharwal, M., Büchi, F. N., Nagashima, S., Marone, F. & Eller, J. Investigation of the transient freeze start behavior of polymer electrolyte fuel cells. *J. Power Sources*. **489**, 229447. 10.1016/j.jpowsour.2020.229447 (2021).

[27] Biesdorf, J., Forner-Cuenca, A., Siegwart, M., Schmidt, T. J. & Boillat, P. Statistical analysis of isothermal cold starts of PEFCs: Impact of gas diffusion layer properties. *J. Electrochem. Soc.* **163**, F1258–F1266. 10.1149/2.1071610jes (2016).

[28] Biesdorf, J., Stahl, P., Siegwart, M., Schmidt, T. J. & Boillat, P. When size matters: Active area dependence of PEFC cold start capability. *J. Electrochem. Soc.* **162**, F1231–F1235. 10.1149/2.0871510jes (2015).

[29] Oberholzer, P. et al. Cold-start of a PEFC visualized with high resolution dynamic in-plane neutron imaging. *J. Electrochem. Soc.* **159**, B235–B245. 10.1149/2.085202jes (2011).

[30] Stahl, P., Biesdorf, J., Boillat, P. & Friedrich, K. A. An investigation of PEFC sub-zero startup: Influence of initial conditions and residual water. *Fuel Cells*. **17**, 778–785. 10.1149/2.0771614jes (2016).

[31] Stahl, P., Biesdorf, J., Boillat, P. & Friedrich, K. A. An investigation of PEFC sub-zero startup: Evidence of local freezing effects. *J. Electrochem. Soc.* **163**, F1535–F1542. 10.1149/2.0771614jes (2016).

[32] Yoshimune, W. et al. Neutron imaging for automotive polymer electrolyte fuel cells during rapid cold starts. *Phys. Chem. Chem. Phys.* **26**, 29466–29474. 10.1039/D4CP03646H (2024). 39576032 10.1039/d4cp03646h

[33] Biesdorf, J. et al. Dual spectrum neutron radiography: Identification of phase transitions between frozen and liquid water. *Phys. Rev. Lett.* **112**, 248301. 10.1103/PhysRevLett.112.248301 (2014). 24996112 10.1103/PhysRevLett.112.248301

[34] Siegwart, M. et al. Distinction between super-cooled water and ice with high duty cycle time-of-flight neutron imaging. *Rev. Sci. Instrum.* **90**, 103705. 10.1063/1.5110288 (2019).

[35] Siegwart, M. et al. Spatially resolved analysis of freezing during isothermal PEFC cold starts with time-of-flight neutron imaging. *J. Electrochem. Soc.* **167**, 064510. 10.1149/1945-7111/ab7d91 (2020).

[36] Higuchi, Y. et al. Pulsed neutron imaging for differentiation of ice and liquid water towards fuel cell vehicle applications. *Phys. Chem. Chem. Phys.* **23**, 1062–1071. 10.1039/D0CP03887C (2021). 33346285 10.1039/d0cp03887c

[37] Isegawa, K. et al. Fast phase differentiation between liquid–water and ice by pulsed neutron imaging with gated image intensifier. *Nucl. Instrum. Methods Phys. Res. Sect. A*. **1040**, 167260. 10.1016/j.nima.2022.167260 (2022).

[38] Higuchi, Y. et al. Experimental visualization of water/ice phase distribution at cold start for practical-sized polymer electrolyte fuel cells. *Commun. Eng.* **3**, 33. 10.1038/s44172-024-00176-6 (2024).

[39] Takahashi, T. et al. Cold start cycling durability of fuel cell stacks for commercial automotive applications. *Int. J. Hydrogen Energy*. **47**, 41111–41123. 10.1016/j.ijhydene.2022.09.172 (2022).

[40] Hu, K. et al. Effect of different control strategies on rapid cold start-up of a 30-cell proton exchange membrane fuel cell stack. *Int. J. Hydrogen Energy*. **46**, 31788–31797. 10.1016/j.ijhydene.2021.07.041 (2021).

[41] Wang, F. et al. Experimental study on rapid cold start-up performance of PEMFC system. *Int. J. Hydrogen Energy*. **48**, 21898–21907. 10.1016/j.ijhydene.2023.01.364 (2023).

[42] Tao, J., Wei, X., Wang, X., Jiang, S. & Dai, H. Control-oriented cold start modelling and experimental validation of PEM fuel cell stack system. *Int. J. Hydrogen Energy*. **50**, 450–469. 10.1016/j.ijhydene.2023.08.240 (2024).

[43] Ma, T. et al. Research on control algorithm of proton exchange membrane fuel cell cooling system. *Energies* **12**, 3692. 10.3390/en12193692 (2019).

[44] Jomori, S., Komatsubara, K., Nonoyama, N., Kato, M. & Yoshida, T. An experimental study of the effects of operational history on activity changes in a PEMFC. *J. Electrochem. Soc.* **160**, F1067–F1073. 10.1149/2.103309jes (2013).

[45] Park, J. et al. Neutron imaging investigation of liquid water distribution in and the performance of a PEM fuel cell. *Int. J. Hydrogen Energy*. **33**, 3373–3384. 10.1016/j.ijhydene.2008.03.019 (2008).

[46] Hwang, G. S. & Weber, A. Z. Effective-diffusivity measurement of partially-saturated fuel-cell gas-diffusion layers. *J. Electrochem. Soc.* **159**, F683–F691. 10.1149/2.024211jes (2012).

[47] Yoshimune, W. & Harada, M. Temperature-induced shear-thinning in catalyst inks. *Electrochem. Commun.* **130**, 107099. 10.1016/j.elecom.2021.107099 (2021).

[48] Yoshimune, W. Dependence of oxygen transport properties of catalyst layers for polymer electrolyte fuel cells on the fabrication process. *Results Chem. 5*. 100738. 10.1016/j.rechem.2022.100738 (2023).

[49] Siemens Solid, E. (2023). https://solidedge.siemens.com/en/ accessed (May 22 2025).

[50] Nonaka, T. et al. Toyota beamline (BL33XU) at SPring-8. *AIP Conf. Proc.* 1741, 030043, (2016). 10.1063/1.4952866

[51] Csoklich, C., Sabharwal, M., Schmidt, T. J. & Büchi, F. N. Does the thermal conductivity of gas diffusion layer matter in polymer electrolyte fuel cells? *J. Power Sources*. **540**, 231539. 10.1016/j.jpowsour.2022.231539 (2022).

[52] Uesugi, K. et al. Development of fast (sub-minute) micro‐tomography. *AIP Conf. Proc.* **1266**, 47–50. 10.1063/1.3478197 (2010).

[53] Schneider, C. A., Rasband, W. S. & Eliceiri, K. W. NIH image to imageJ: 25 years of image analysis. *Nat. Methods*. **9**, 671. 10.1038/nmeth.2089 (2012). 22930834 10.1038/nmeth.2089 PMC5554542

[54] GeoDict - The Digital Material Laboratory. May (2025). https://www.geodict.com/ accessed 22.

[55] Zenyuk, I. V., Parkinson, D. Y., Connolly, L. G. & Weber, A. Z. Gas-diffusion-layer structural properties under compression via X-ray tomography. *J. Power Sources*. **328**, 364–376. 10.1016/j.jpowsour.2016.08.020 (2016).

